# Establishment of an Experimental Procedure for Preparing Trial Serum Samples for the Specific Serodiagnosis of *Toxocara canis* for External Quality Assessment Schemes

**DOI:** 10.1155/2020/6842975

**Published:** 2020-02-10

**Authors:** Quang Huy Vu, Diep Tuan Tran, Phu Manh Sieu Tran, Van Chuong Le, Thi Diem Phuc Huynh, Quang Sang Bui

**Affiliations:** ^1^University of Medicine and Pharmacy at Ho Chi Minh City, Vietnam; ^2^University Medical Center Ho Chi Minh City, Vietnam; ^3^Quality Control Center for Medical Laboratory under Supervision of the Ministry of Health, University of Medicine and Pharmacy at Ho Chi Minh City, Vietnam; ^4^Nguyen Trai Hospital, Ho Chi Minh City, Vietnam

## Abstract

**Background:**

External quality assessment (EQA) provides evidence of reliable, accurate, and precise results for customers using the diagnostic test for *Toxocara canis. Objective*. To establish a procedure for producing standard *Toxocara canis* serum samples for serodiagnostic testing in EQA.

**Methods:**

The collected serum samples to contain anti-*Toxocara canis* serum samples for serodiagnostic testing in EQA. *F*-test and a *T*-test were applied to evaluate their homogeneity and stability.

**Results:**

Among eleven samples positive by ELISA, ten of them were confirmed via Western blotting by positive reaction with 5 specific *Toxocara canis* serum samples for serodiagnostic testing in EQA. *Toxocara canis* serum samples for serodiagnostic testing in EQA. *p* > 0.05). Samples produced by both methods were stable for 7 days at 30°C (*p* > 0.05). Samples produced by both methods were stable for 7 days at 30°C (

**Conclusion:**

Specific serodiagnosis samples of anti-*Toxocara canis* antibodies for EQA could be produced that possessed homogeneity and stability lasting for 3 months and 6 months by the freeze-drying and freezing methods, respectively. At 30°C, the samples produced by both methods were stable for 7 days, suitable for delivery to remote laboratories.*Toxocara canis* serum samples for serodiagnostic testing in EQA.

## 1. Introduction

Human toxocariasis is described to be one of the most common public and economically critical zoonotic parasitic disease caused by infection with larvae of *Toxocara canis* and *Toxocara cati* [1]. Human beings acquire toxocariasis through a range of routes, such as accidental ingestion of infective eggs from contaminated soil, water, raw vegetables, or fruit. Most infections are asymptomatic; two distinct clinical syndromes are classically recognized: visceral larva migrant (a systemic disease caused by larval migration through various major organs, including the lungs, liver, muscles, and CNS) and ocular larva migrants (a disease limited to the eyes and optic nerves) such as uveitis. The global prevalence of *T. canis* infections or exposure in human beings, as determined using serological assays, although still fragmentary, varies from 1.6 to 87 percentage [1]. The prevalence of anti-*T. canis* serum antibody has been reported at 45.2% in the southern part of Vietnam in 2012 [2]. Beside serological or immunological methods, the diagnosis of toxocariasis and Toxocara infection can be performed by histopathological examination, morphometric assessment of larvae (if present), or the specific detection of larval DNA from tissue or body fluid sample. Among them, biopsy and visual detection of parasite is identified a gold standard. However, this method is extremely invasive and depends on the larval load and the stage of the infection [3]. Therefore, many serological methods have been developed and widely applied in a clinical approach. Nevertheless, the sensitivity and specificity of serological and immunological assays depend on both the antigens (e.g., crude products from *T. canis* larvae, native or recombinant *Toxocara* spp. excretory-secretory (TES) antigens, or either glycan antigens or deglycosylated TES antigens) and the type of antibodies (e.g., total IgG, IgG subclass, or IgM) that are being measured [1]. The use of the TES antigens in ELISA has long been utilized as a standard immunological method. Nevertheless, the antibodies generated against other helminthic infections can cause cross-reaction to native TES antigens [4]. Thus, the specificity of serological assays is extremely important. To eliminate the false positive result, the confirmation by Western blotting is required [3].

EQA is one of the critical elements of a laboratory quality management system, in accordance with ISO 15189:2012 [5]. In addition, EQA provides objective evidence of reliable, accurate, and precise results for all customers using the services of the laboratory. The specific serodiagnosis of anti-*Toxocara canis* antibodies for EQA was designed to improve the quality of screening and diagnostic tests for *Toxocara canis*, which plays a key role in the control and evaluation of the quality of a laboratory via interlaboratory comparisons. EQA participation is vital for all medical laboratories [6]. A report on the quality of laboratories performing serological diagnosis of Toxoplasma sp. was performed from 2004 to 2013 by the National Center for Clinical Laboratories in China. The results were 5384 EQA test reports for Toxoplasma-specific IgM and 2666 EQA test reports for Toxoplasma-specific IgG. The IgM detection ranged between 84.3 and 99.6%; IgG detection ranged between 61.1 and 99.3%. The most common problem was failure to detect low titers of antibody [7].

Ideal samples for an EQA program would satisfy a range of criteria: stable for the conditions under which they will be transported and stored, homogeneous across all the aliquots produced, have analyte concentrations that include the expected clinical range, include appropriate sample types (e.g., urine, whole blood, and serum), available in sufficient volume, inexpensive enough for cost not to be an impediment, and behave in clinical laboratory measurement procedures in the same manner as patient samples. In practice, it is impossible to achieve all these goals, and some compromises are required in the preparation of EQA materials [8]. To date, no study on the production of trial samples and launching EQA program for the specific serodiagnosis of anti-*Toxocara canis* antibodies in Vietnam has been reported. Therefore, we developed a procedure for producing standard samples that contain specific anti-*Toxocara canis* antibodies for use in EQA.

## 2. Materials and Methods

### 2.1. Sample Collection

This study was conducted on serum samples collected from donors who have been in close contact with dogs or related species and have shown some specific toxocariasis symptoms [9–11]. These samples have shown positive reactions with specific IgG anti-*Toxocara canis* antibodies at the Institute of Malaria Parasitology and Entomology, Binh Dinh Province, Vietnam.

From September to December 2017, the collected serum samples that met the designed inclusion criteria were extracted into Eppendorf tubes and stored at -20°C. All of the samples were transported to the Quality Control Center for Medical Laboratory under the Ministry of Health, University of Medicine and Pharmacy at Ho Chi Minh City, for the subsequent steps in the procedure.

Hemolyzed samples or sera that have turned dark after 48 hours of storage were excluded from this study. To ensure the homogeneity of the serum samples, all samples were kept at -20°C during transportation and storage. The samples were tested by both *Toxocara canis* IgG ELISA (NovaTec Immundiagnostica GmbH, Germany) and *Toxocara* Western blotting IgG (LDBIO Diagnostics, Lyon, France) to confirm the presence of specific IgG anti-*Toxocara canis* antibodies [12]. To ensure that the donors were not infected with other helminths, which have high prevalence in Vietnam [13, 14], as well as to prevent potential cross-reactions, the samples were tested by using the ELISA technique and found to be negative for other helminth antibodies, including *Taenia solium* IgG, *Echinococcus IgG*, *Fasciola* IgG, *Paragonimus westermani* IgG, and *Clonorchis sinensis* IgG (Creative Diagnostics, New York, USA) [15]. All samples that were negative for antibodies to HIV-1, HIV-2, HCV, and HBV were selected for further analysis.

### 2.2. The Procedure

The trial samples were divided into two lots and then stored in different conditions. The freezing method samples were stored at -80°C and 2-8°C, and the freeze-drying method samples were also stored at -80°C and 2-8°C. Each lot contained 100 tubes, with 100 *μ*L of sample per individual tube.

The methods for testing the homogeneity and stability were in compliance with ISO Guide 35 and ISO 13528 [16]. Ten tubes from each lot were selected randomly. The ELISA assay, read at a wavelength of 450 *η*m, was used to evaluate homogeneity. The absorbance values were converted into NovaTec Units (NTU) using the following formula as specified by the manufacturer: patient samples (mean) absorbance value × 10/mean absorbance value of the cut − off controls = NTU (>11 NTU was considered as positive as specified by the manufacturer). The stability was assessed after 1 month, 3 months, and 6 months by randomly testing three tubes from each lot to determining the NTU by ELISA and to calculate the average of NTU values at the corresponding time points.

### 2.3. Data Analysis

The *F*-test (one-way ANOVA) was carried out to evaluate the homogeneity of the samples (*F* statistics < *F* distribution). The *T*-test (independent-sample *t*-test), sig (2 − tailed) > 0.05, at *α* = 0.05, allowed us to evaluate the stability of the samples. SPSS 20.0 software (IBM, New York, USA) was used for data analysis.

## 3. Results

### 3.1. Anti-Toxocara canis-Specific Antibody Assessment

**Figure 1:**
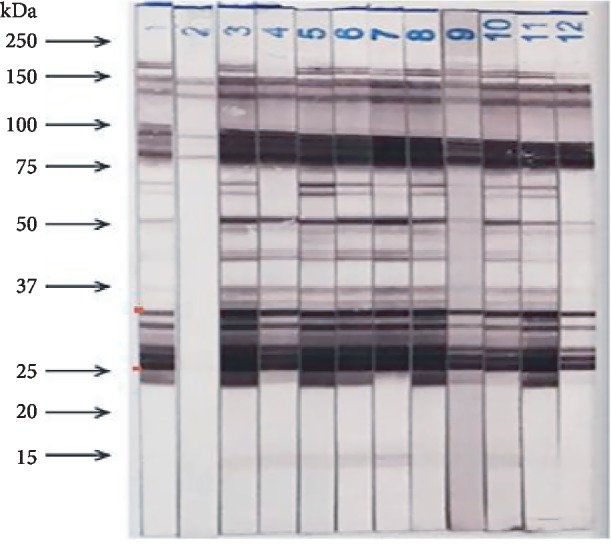
Protein bands detected by Western blotting that correspond to *Toxocara canis* antigens. Strips 1 and 2 were the positive and negative controls, respectively, while strips 3 to 12 represent positive serum samples.

Eleven serum samples were found to be positive for anti-*Toxocara canis* antibodies and having high concentrations of the antibodies, as determined by ELISA: the concentration of the lowest sample was 31.52 NTU, and the concentration of the highest sample was 56.58 NTU (Table 1). The specific IgG antibodies against *Toxocara canis* antigens were found to be positive for 5 bands between 24 and 35 kDa that were specific to *Toxocara canis* by the Western blotting technique (Figure 1). However, sample number 2 had a negative result. None of the samples were positive for antibodies against other helminths except the sample 9 (Table 2) or against HIV, Hepatitis B Virus, or Hepatitis C Virus.

Only sample No. 9 showed a reaction with *Fasciola* IgG, while the other samples showed reactions with *Toxocara canis* IgG and no reaction to other helminths (Table 2).

Among these eleven samples, strip numbers 3-12 showed positive reactions in both ELISA and Western blotting, while strip number 2 showed a positive ELISA result and a negative Western blotting result; strip number 1 represents a positive control sample (Figure 1).

The serum samples that reacted with the 5 bands between 24 and 35 kDa in the Western blotting, confirming that they contained specific anti-Toxocara antibodies, were divided into microcentrifuge tubes and processed by freezing or freeze-drying. This produced two lots of trial samples containing IgG antibodies specific to *Toxocara canis*:

- Lot 1: samples were produced by the freeze-drying method (100 tubes), with each tube containing 100 *μ*L of serum sample with a concentration of 31.01 NTU
- Lot 2: samples were produced by the freezing method (100 tubes), with each tube containing 100 *μ*L of serum sample with a concentration of 27.18 NTU

### 3.2. Evaluation of Homogeneity

Table 3 shows the assessment results of the homogenous samples DK and DL. The samples produced by the freezing method (DL) had an *F*_sig_ of 2.52, less than the *F*_*α*_ of 3.02. The samples produced by the freeze-drying method (DK) had an *F*_sig_ of 0.59, less than the *F*_*α*_ of 3.02 (Table 3). We assumed that the *H*_0_ hypothesis was accepted. This finding indicates that the trial samples for EQA that were produced by both the freeze-drying and freezing methods were homogeneous.

### 3.3. Evaluation of Stability

#### 3.3.1. The Long-Term Stability of the Serum Samples

When stored at -80°C, the freeze-dried samples were stable for at least 3 months and the freezing samples were stable for 6 months (*p* > 0.05). The samples produced by both methods were stable for 7 days at 30°C (*p* > 0.05).

Table 4 shows that the serum samples produced by freeze-drying were stable for 1 month when stored at -80°C, with a *p* > 0.05. The serum samples produced by freezing were stable for 6 months when stored at -80°C, with a *p* > 0.05. The antibody concentration decreased after 6 months. At -80°C, the freeze-dried samples had concentrations of 31.87 NTU at 1 month, 28.48 NTU at 3 months, and 28.02 NTU at 6 months, while the freezing samples had concentrations of 27.3 NTU at 1 month, 26.79 NTU at 3 months, and 26.44 NTU at 6 months (Table 4). These results showed that the freezing samples (DL) were stable for 6 months.

At 30°C, the serum samples produced by the freeze-drying and freezing methods were stable for 7 days (Table 5).

#### 3.3.2. Results on the Production of the Trial Serum Samples Containing an Anti-Toxocara canis Antibody for EQA

**Figure 2:**
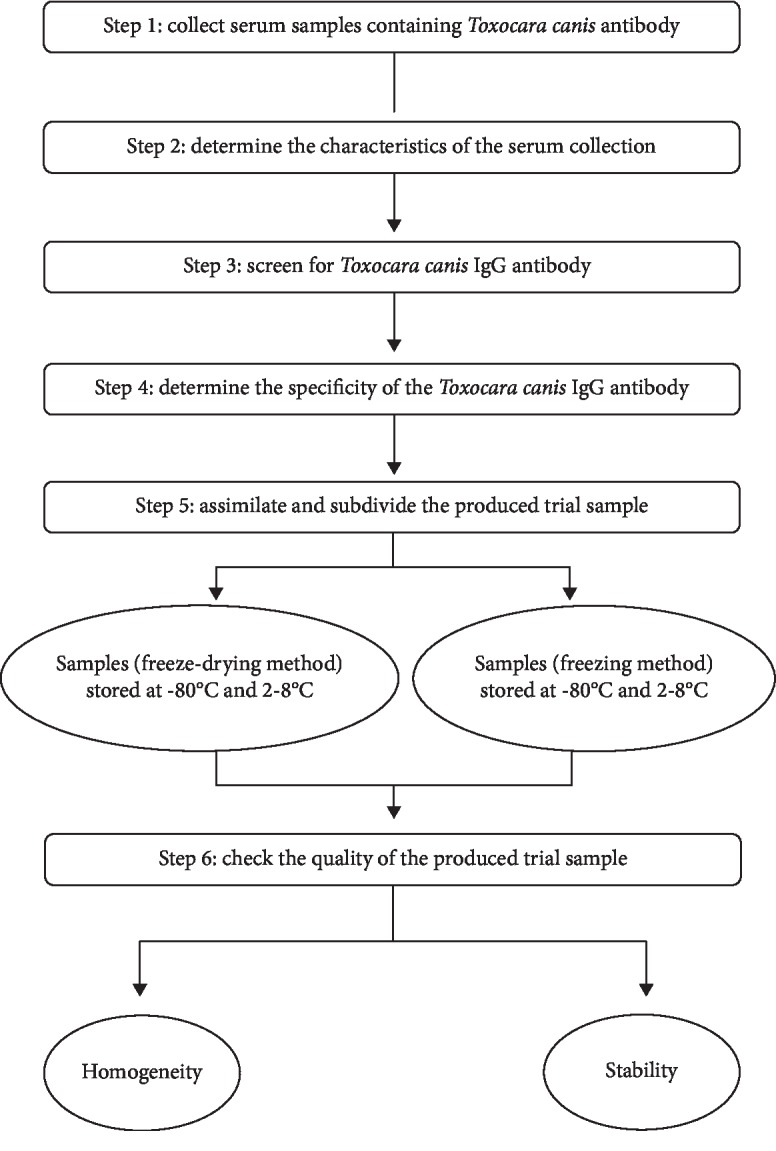
The workflow of preparation of trial samples containing an anti-*Toxocara canis* antibody used in EQA.

The procedure for production of the samples for the specific serodiagnosis of anti*-Toxocara canis* antibodies via an external quality assessment scheme has been developed (Figure 2).

## 4. Discussion

### 4.1. Determination of the Quality of the Trial Samples Containing an Anti-Toxocara canis Antibody Used in EQA

#### 4.1.1. The Trial Samples

The process of manufacturing serum containing anti-*Toxocara canis* antibodies started from the collection of serum samples taken from patients without the addition of preservatives. Human serum was the best option, but this was recommended only in cases when animal serum was unsuitable. The immune responses to *Toxocara* sp. in dogs, cats, rabbits, and humans are completely different [17–19]. In this study, serum was taken from patients. We did not use mixed sera, as mixing might affect the specificity of the anti-*Toxocara canis* antibodies. The mixing should be performed when all of the samples are known to contain anti-*Toxocara canis-*specific antibodies, which will increase the cost of production. The original yellow serum did not contain blood cells and was not hemolyzed or cloudy. The serum samples with a concentration of 10 mg/mL hemoglobin, 5 mg/mL triglyceride, and 0.2 mg/mL bilirubin might interfere with ELISA results. The samples were screened and found to be negative for antibodies to HbsAg and for anti-HCV and anti-HIV-1/2 antibodies. However, these samples must be handled as potentially infectious products.

All ten serum samples containing anti-*Toxocara canis* antibodies identified by ELISA reacted with 5 bands between 24 and 35 kDa in the Western blotting analysis. These bands, grouped and well isolated, were characteristic and generally easily observable [20, 21]. Therefore, the assessment of the specificity of anti-*Toxocara canis* antibodies in the original serum was necessary when manufacturing serum to be used for *Toxocara canis* external quality assessment.

The next stages were implemented in accordance with ISO/IEC 17043:2011 [22] and ISO 13528: 2015 [16].

### 4.2. The Homogeneity and Stability of Samples Produced by Freeze-Drying and Freezing Methods

Freeze-drying and freezing are two methods used in manufacturing the samples to be used for external quality assessment worldwide. Serum samples produced by freeze-drying and freezing were found to be homogeneous by the Fisher tests. This was reasonable, as our sample materials were sera taken from patients without the addition of preservatives, thus retaining the same substrates and ensuring homogeneity. Samples produced by freezing were more homogeneous than those produced by freeze-drying. Samples produced by the freeze-drying method had a lower homogeneity, which might be due to the effects of freeze-drying and reconstitution. Samples produced by freezing were more stable than those produced by freeze-drying: hydrogen bonds in water contributed significantly to the stability of protein structure. In this case, in the freeze-drying method, the removal of water tended to cause the physical instability of proteins. Furthermore, even after successful freeze-drying, the protein structures might be influenced by factors including synthesis, oxidation, the Maillard reaction, and hydrolysis. Therefore, the long-term stability of the freeze-drying method might still be limited, especially at high temperatures. These problems could be minimized by choosing the optimal pH and remaining moisture content, and more importantly, by the addition of stabilizers that might prevent tissue freezing and cell destruction during the cooling processes [23].

### 4.3. The Process of Production of Trial Samples Containing Anti-Toxocara canis Antibodies Used in EQA

The procedure of production of the trial samples has been completed. This procedure can be used for the mass production of standard serum samples that contain anti-*Toxocara canis* for EQA in the future. The whole procedure satisfied the requirements of a laboratory biosafety manual [24]. By utilizing Western blotting in the 4^th^ step, the specificity of anti-*Toxocara canis* antibodies in the serum samples was increased. ISO/guide 35:2017 and ISO 13528: 2015 were the criteria we followed when evaluating the homogeneity and stability of the samples. Therefore, the quality of the produced samples was preserved during storage time.

## 5. Conclusion

1. The specific IgG antibodies against *Toxocara canis* antigens in the collected serum samples were found to be reactive with 5 bands between 24 and 35 kDa in the Western blotting analysis
2. Samples produced by the freezing method were more homogenous and stable than those produced by the freeze-drying method: the freeze-drying samples were stable for 3 months, while the freezing samples were stable for 6 months at -80°C. At 30°C, the trial samples produced by both methods were stable for 7 days
3. The process of preparation of the trial EQA samples of anti-*Toxocara canis* antibodies can be used to produce serological EQA samples for the assessment of parasitosis

## Figures and Tables

**Table 1:** Results of the anti-*Toxocara canis*-specific antibody assessment.

No	ELISA				Western blotting
	Cut-off	OD	NTU	Result	
1	QC				Positive
2	0.547	2.18	39.82	Positive	Negative
3	0.547	3.04	55.54	Positive	Positive
4	0.547	2.61	47.69	Positive	Positive
5	0.547	2.86	52.36	Positive	Positive
6	0.547	3.09	56.58	Positive	Positive
7	0.547	3.04	55.56	Positive	Positive
8	0.547	2.99	54.63	Positive	Positive
9	0.547	1.72	31.52	Positive	Positive
10	0.547	2.34	42.79	Positive	Positive
11	0.547	1.91	34.91	Positive	Positive
12	0.547	2.56	46.78	Positive	Positive

**Table 2:** ELISA reaction results to *Toxocara canis* and helminth antigens.

Helminth antigens	Sample no.	No. (%) of positive samples	OD (mean ± SD)
*Toxocara canis* IgG	2, 3, 4, 8, 6, 7, 8, 10, 11, 12	11 (100)	2.74 ± 0.5
*Clonorchis sinensis* IgG		0	0
*Paragonimus westermani* IgG		0	0
*Taenia solium* IgG		0	0
*Fasciola* IgG	9	1 (9.1)	2.09
*Echinococcus* IgG		0	0

**Table 3:** Evaluating the homogeneity of the freeze-dried and freezing method samples.

Index	DK (freeze-dried samples)	DL (freezing samples)
Mean ± SD	31.01 ± 1.1	27.18 ± 0.9
CV (%)	3.7	3.7
*F*_sig_	2.52	0.59
*F*_*α*_	df1 = 9, df2 = 10, *α* = 0.05 then *F*_*α*_ = 3.02	

**Table 4:** Results of the NTU values during the study period.

Time	Index	DK (freeze-dried samples)		DL (freezing samples)	
		Stored at -80°C	Stored at 2-8 °C	Stored at -80°C	Stored at 2-8°C
1 month	Mean ± SD	31.87 ± 1.4	28.85 ± 1.2	27.3 ± 0.3	26.08 ± 0.9
	CV (%)	4.4	4.1	1.2	3.5
	*p* value	>0.05	<0.05	>0.05	<0.05

3 months	Mean ± SD	28.88 ± 1.1	25.43 ± 0.7	26.8 ± 0.3	24.56 ± 1.1
	CV (%)	3.9	2.8	1.2	4.5
	*p* value	>0.05	<0.05	>0.05	<0.05

6 months	Mean ± SD	28.02 ± 1.1	23.86 ± 0.7	26.44 ± 0.3	24.0 ± 1.0
	CV (%)	3.9	2.8	1.2	4.4
	p-value	<0.05	<0.05	>0.05	<0.05

**Table 5:** Stability of the serum samples at 30°C.

Time	Index	DK (freeze-dried samples)	DL (freezing samples)
5 days	Mean ± SD	25.47 ± 0.7	26.26 ± 1.0
	CV (%)	2.6	3.9
	*p* value	>0.05	>0.05

7 days	Mean ± SD	25.5 ± 0.7	26.31 ± 1.1
	CV (%)	2.6	3.9
	p-value	>0.05	>0.05

## Data Availability

The data used to support the findings of this study are included within the article.

## Abbreviations

ELISA: Enzyme-linked immunosorbent assay
EQA: External quality assessment
HBs: Hepatitis B surface
HBV: Hepatitis B virus
HCMC: Ho Chi Minh City
HCV: Hepatitis C virus
HIV: Human immunodeficiency virus
ISO/IEC: International Standard Organization/International Electrotechnical Commission
NTU: NovaTec Units
SPSS: Statistical Package for Social Sciences.
